# Changes in diet from pregnancy to one year after birth: a longitudinal study

**DOI:** 10.1186/s12884-021-04038-3

**Published:** 2021-09-04

**Authors:** Tanja Poulain, Ulrike Spielau, Mandy Vogel, Anne Dathan-Stumpf, Antje Körner, Wieland Kiess

**Affiliations:** 1 LIFE Leipzig Research Center for Civilization Diseases, Leipzig University, Philipp-Rosenthal-Strasse 27, 04103 Leipzig, Germany; 2 Department of Women and Child Health, University Hospital for Children and Adolescents and Center for Pediatric Research, Leipzig University, Liebigstrasse 20a, 04103 Leipzig, Germany; 3 Department of Obstetrics and Gynecology, University Hospital Leipzig, Liebigstrasse 20a, 04103 Leipzig, Germany

**Keywords:** Diet, Longitudinal, Pregnancy, Breastfeeding, Weaning

## Abstract

**Background:**

Pregnancy and the first year after giving birth are marked by physiological and psychological changes. While it is well known that energy requirements change during this time, the question of how a woman’s diet actually changes from pregnancy until 1 year postpartum has been left virtually unexplored. The present study employs a longitudinal design to investigate these changes.

**Methods:**

Data were collected within the framework of the LIFE Child study (Leipzig, Germany). The diet composition and culture of eating of 110 women were assessed at 3 time points: in the 24th week of pregnancy, 3 months after giving birth (breastfeeding period), and 12 months after giving birth (after weaning). We assessed differences in nutritional health (Nutritional Health Score, NHS) and the consumption of different food items at each of these time points. We also investigated associations between nutritional health and age, socio-economic status (SES), BMI before pregnancy, and previous births at all three time points.

**Results:**

The analyses revealed high correlations in the NHS values between the three time points (rho_t0/t1_ = .55, rho_t0/t2_ = .60). On average, nutritional health was lower in the breastfeeding period than during pregnancy. In more detail, women reported less healthy levels of treats and white bread consumption and a higher frequency of snacking in the breastfeeding period than during pregnancy. In contrast, overall nutritional health did not differ significantly between pregnancy and the time after weaning. Increased age was associated with a healthier diet during pregnancy, and a high SES was associated with healthier diet after weaning. Furthermore, the increase in nutritional health from the breastfeeding period to the time after weaning was significantly stronger in women with a higher BMI. We observed no significant associations between dietary nutritional health and previous births.

**Conclusions:**

The present findings suggest that higher energy requirements in the breastfeeding period are met by consuming high-calorie and unhealthy food products rather than healthy and nutrient-rich food. Young mothers should be supported in taking care of their own nutritional health during the challenging time of breastfeeding and caring for a newborn child.

**Supplementary Information:**

The online version contains supplementary material available at 10.1186/s12884-021-04038-3.

## Background

A healthy diet promotes healthy development at all stages of life. A healthy diet is particularly important during pregnancy and breastfeeding, however, not only for the (expectant) mother, but also for the (unborn) child. A healthy diet before or during pregnancy, for example, has been associated with a lower risk of gestational diabetes [1, 2] and a healthier birthweight [3, 4], and healthy nutrition during breastfeeding has been associated with beneficial metabolic development in infants [5]. A relevant consideration is that, for the mother, the periods of pregnancy and the first year after giving birth are characterized not only by hormonal changes [6] but also by new emotional experiences [7, 8]. Diet can also change during this time, for instance, due to specific recommendations (e.g., avoiding certain raw ingredients during pregnancy and breastfeeding [9]), changing tastes [10, 11], self-imposed food restrictions [12], or changing demands on time.

According to the European Food Safety Authority, the average additional energy requirement during pregnancy is 70 kcal/day in the 1st trimester, 260 kcal/day in the 2nd trimester and 500 kcal/day in the 3rd trimester [13]. The additional energy requirement during breastfeeding is estimated at 500 kcal/day, the same as in the last trimester of pregnancy [13]. However, in a more general sense, it is important that the diet during these periods is based on recommendations for healthy adults, e.g., plenty of fruit and vegetables and limited consumption of fatty or sweet foods [9].

Studies indicating increased consumption of sugary and fatty foods and infrequent success in meeting recommended daily serving guidelines during pregnancy and breastfeeding/postpartum [14, 15] suggest that additional energy requirements in these periods are more likely to be satisfied by consuming unhealthy food. This runs contrary to the recommendations for a healthy, balanced diet, especially during pregnancy and breastfeeding [9]. Factors that may have a further negative impact on diet during pregnancy and breastfeeding include higher body mass index (BMI) [16, 17], lower socioeconomic status (SES) [16–18], and lower age of women [16, 18]. It is also possible that a mother’s diet during or after pregnancy is affected by whether she has had other children. On the one hand, first-time mothers may feel less secure in their role as mothers. On the other hand, the absence of older children who also require their attention could mean they can devote more time and attention to their own diet. Potentially, insecurity and additional time burdens could have a negative impact on the mother’s nutrition. However, a previous study investigating the diet of first-time and second-time mothers reported no meaningful differences between these groups [15].

Despite a wide range of research into nutrition during pregnancy and breastfeeding, the question of how mothers’ nutrition actually changes during the period from pregnancy to the end of breastfeeding has been virtually unexplored. For this reason, one aim of the project discussed below was to assess and compare women’s diet composition at three points in time, specifically the 24th week of pregnancy (pregnancy), a few months after giving birth (breastfeeding period), and approximately 1 year after giving birth (after weaning). A further aim of the study was to investigate possible effects of age, SES, BMI before pregnancy, and previous births on the healthiness of the mother’s diet at these different points in time.

## Methods

### Participants

**Fig. 1:**
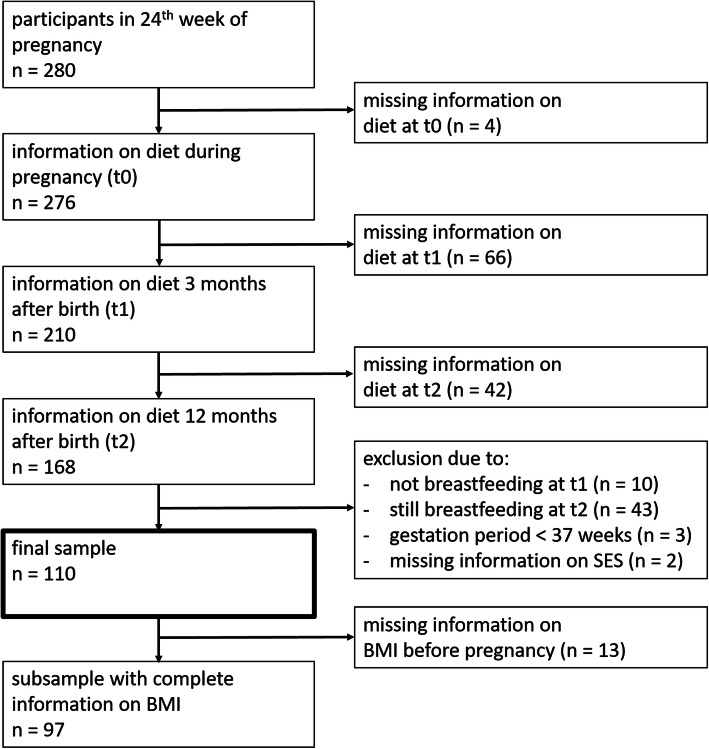
Study sample and excluded cases

Data for the project were collected between 2016 and 2020 as part of the LIFE Child study, an ongoing German child cohort study examining health and development in pregnant women and in children and their parents [19, 20]. Expectant mothers were mainly recruited by advertising at different institutions such as centers for prenatal diagnostics or gynecologist practices. Data for all women who completed the questionnaire on diet composition and culture of eating (CoCu) at three specific time points were eligible for inclusion in the present study. These time points were a) the first study visit during pregnancy, specifically the 24th week of pregnancy (t0, pregnancy), b) the study visit scheduled 9–17 weeks after delivery, when the child is approximately 3 months old (t1, breastfeeding period), and c) the study visit scheduled 48–56 weeks after delivery, when the child is approximately 12 months old (t2, after weaning). Of 280 individuals who had participated at t0 since May 2016 (when CoCu was introduced at LIFE Child) and could potentially attend an appointment at t2 by spring 2020 (when data analysis began), 168 completed the questionnaire at all three time points (see Fig. 1). Of these women, those not breastfeeding at t1 (*n* = 10), those still breastfeeding at t2 (*n* = 43), those with a gestation period of less than 37 weeks (*n* = 3), and those with missing information on SES (*n* = 2) were excluded from further analyses. The final sample comprised 110 women aged between 23 and 40 years (mean = 32.2). Information on BMI before pregnancy was missing for 13 of these women, resulting in a reduced number of women being included in the analysis of potential associations between nutritional health and BMI before pregnancy (*n* = 97, see Fig. 1).

The LIFE Child study was designed in accordance with the Declaration of Helsinki and was approved by the Ethics Committee of the Medical Faculty at the University of Leipzig (Reg. No. 264/10-ek). All participants were informed regarding the content of the study and provided informed written consent before participating.

### Measures

#### CoCu: diet composition and culture of eating

CoCu is a short nutritional questionnaire that assesses diet composition and culture of eating in children and adults [18, 21]. The questionnaire was designed by the authors for use in the LIFE Child cohort study, with the main goal of assessing the nutritional quality of the subject’s diet as quickly and efficiently as possible. Within LIFE Child, the questionnaire is used to assess the diets of children (at each study visit), parents (at each study visit), and pregnant women (in the 24th week of gestation).

The CoCu questionnaire consists of two parts, a diet composition part and a culture of eating part. In the diet composition part, participants indicate what they eat, i.e., how many portions of different food products they consume per day (for fruits/vegetables, unsweetened milk products, sweetened milk products, sweetened beverages, whole grain bread, white bread) or per week (for meat, fish, ready-made meals, fried potatoes, potatoes, rice/noodles, cakes, sweet or savory snacks). The selection of items was primarily based on the food groups included in a Food Frequency Questionnaire (FFQ) [22], with a single food item chosen to represent each food group. Answers are given on a 6-point scale ranging from 1 (“never”) to 6 (“> 7 portions”). To test the validity of the questionnaire, responses to questions on the diet composition part of CoCu were compared with responses to questions in the FFQ in a sample of 105 young people aged between 13 and 19 years [21] and in a sample of 430 pregnant women [18]. Levels of association between the CoCu and FFQ responses varied across the questions but were broadly satisfactory [18, 21]. For further analyses, the level of consumption of each food product can be categorized as either healthy (“green” or “10 points”), moderately healthy (“yellow” or “0 points”), or unhealthy (“red” or “-10 points”) (see Additional file 1), based on German dietary guidelines [23]. For the purpose of categorization, three of the original food items (sweetened milk products, cakes, and sweet or savory snacks) are aggregated into one item, described as “treats”. By summing the categorizations/points of all food items, a “Nutritional Health Score” (NHS) can be derived [21]. This score ranges between − 120 (in cases where all responses were categorized as unhealthy) and + 120 (in cases where all responses were categorized as healthy).

The culture of eating part of the CoCu questionnaire contains questions on how participants eat, e.g., on specific diets, on the number of meals per day, on snacking between meals, and on the use of media while eating. More details on the questionnaire development, validity and reliability are provided elsewhere [18, 21].

In the present project, all questions of the diet composition part and a selection of items of the culture of eating part, specifically regarding the number of meals per day (first breakfast, second breakfast, lunch, afternoon snack, dinner) and snacking between meals, were included in the analyses (see Additional file 2).

#### Socio-economic status

In the present project, information on the SES of the participants’ families was collected via questionnaires completed at t0. Participants provided information on their own and their partner’s education and occupational position and on the equivalent household income. Using this information, we derived a composite SES score. This score ranges between 3 and 21, with higher scores indicating higher SES. Based on cut-offs provided in a large population-based German study, the score can be categorized into the categories lower SES, middle SES, and high SES [24, 25]. In a representative sample, 60% of the participants would be expected to have a middle SES, 20% a low SES, and the remaining 20% a high SES [24].

#### BMI before pregnancy and previous births

In Germany, information on weight and height before pregnancy and the number of and outcomes of previous births (all reported by the pregnant women) is documented by the treating gynecologist. This information was used in the present analyses. For descriptive purposes, BMI was divided into weight groups based on the following cut-offs [26]: underweight: BMI < 18.5, normal weight: BMI 18.5–24.9, overweight: BMI 25–29.9, obese: BMI > 30.

#### Data analysis

Correlations between the NHS at the different time points were assessed using Spearman correlations.

For the assessment of differences in the healthiness of the participants’ diets between the time points, we dichotomized the consumption levels of the different food items into “healthy” (= reaching the “green” category) and “not healthy” categories. The respective differences between time points were examined by applying logistic generalized estimating equation models (GEE). The same method was applied for the comparison of levels of snacking between meals. For comparisons regarding NHS and the number of meals per day, we applied linear GEE models.

In order to assess whether or not the NHS at t0, t1 and t2 (dependent variable) was associated with age, SES (middle versus high), BMI before pregnancy, or previous births (independent variables), we applied linear GEE models. To assess associations between differences in nutritional health at the different time points with the same cofactors, we applied univariate linear regression analyses. In the case of a significant univariate association (determined by *p* < .05), we tested whether the association remained significant after adjusting for the other confounders that showed a significant association with NHS.

For all GEE models, we assumed an auto-regressive correlation structure between the observations at the different time points. The effects of the independent variables were modeled nested within the time points and reported as odds ratios (logistic GEE) and slopes (linear GEE).

## Results

### Data description

Characteristics of the participating women are presented in Table 1.

**Table 1:** Description of the sample of participating women (*n* = 110)

**Socio-demographics**		
Age at t0 (pregnancy)	mean (sd, range)	32.9 (4.0, 24–41)
SES	n (%) middle	58 (53%)
	n (%) high	52 (47%)
School degree	n (%) highest (German “Abitur”)	89 (81%)
	n (%) lower	16 (15%)
	n (%) missing	5 (4%)
**BMI before pregnancy**		
BMI	mean (sd, range)	23.3 (5.19, 16.4–53.5)
Weight status^a^	n (%) underweight	6 (6%)
	n (%) normal weight	73 (66%)
	n (%) overweight	10 (9%)
	n (%) obese	8 (7%)
	n (%) missing	13 (12%)
**Pregnancy**		
Week of gestation	n (%) 37–38	16 (15%)
	n (%) 39–40	73 (66%)
	n (%) 41	21 (19%)
Previous pregnancies	n (%) yes	55 (50%)
Previous births	n (%) yes	39 (35%)
Complications pregnancy^b^	n (%) yes	29 (26%)
**Residency**		
Residency	n (%) urban	87 (79%)
	n (%) suburban/rural	18 (16%)
	n (%) missing	5 (5%)

^a^Weight groups were categorized based on the following cut-offs [26]: underweight: BMI < 18.5, normal weight: BMI 18.5–24.9, overweight: BMI 25–29.5, obese: BMI > 30

^b^Complications may include general disease, long-term medication, mental stress, bleeding, placenta previa, multiple births, hydramnion, oligohydramnios, uncertainties regarding date of birth, placental insufficiency, cervical weakness, premature labor, anemia, urinary tract infection, positive indirect Coombs test, abnormal serum findings, protein excretion 1%, hypertension, hypotension, edema, gestational diabetes, adjustment anomaly, or others

**Fig. 2:**
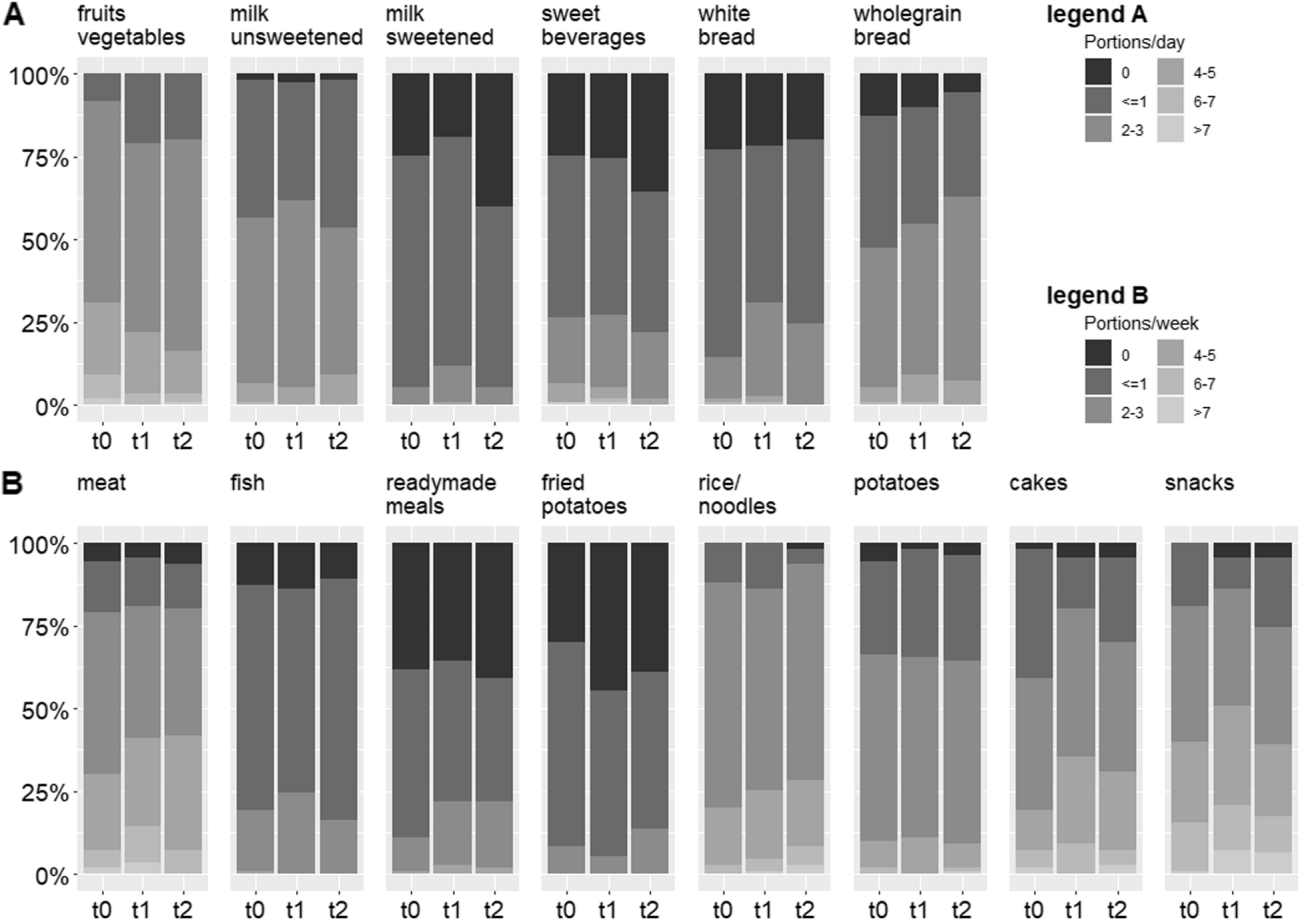
Consumption of different food items at t0 (pregnancy), t1 (breastfeeding), and t2 (after weaning), expressed as portions per day (A) or portions per week (B)

The numbers of portions consumed daily or weekly for each food product at t0, t1, and t2 are shown in Fig. 2. Fruits and vegetables were consumed most frequently. The participants also reported high consumption of whole grain bread and milk products. Ready-made meals, fried potatoes, and fish were consumed least frequently. The percentages of participants whose consumption of the different food items was categorized as “healthy” (“green” category, see Additional file 1) at t0, t1, and t2 are presented in Table 2. This percentage was highest (82–89%) for fish and rice/noodles, and lowest (16–22%) for fruits/vegetables (at t1 and t2) and treats (at t1). The median of the NHS at t0, t1, and t2 were 50 (IQR = 32.5–67.5), 40 (IQR = 30–60), and 50 (IQR = 30–70), respectively, indicating that the ratio of healthy to unhealthy diets was positive at all time points.

**Table 2:** Percentages of healthy consumption levels by time point (*n* = 110)

		Percentage of healthy consumption			*p*-values for differences in percentages		
		T0	T1	T2	T0/T1	T1/T2	T0/T2
**Diet composition**							
Fruits/vegetables	% healthy	31%	22%	16%	.057	.178	.003
Milk unsweetened	% healthy	50%	56%	45%	.310	.022	.400
Beverage sweetened	% healthy	25%	25%	36%	.847	.020	.022
Whole grain bread	% healthy	42%	46%	56%	.505	.068	.021
White bread	% healthy	86%	69%	76%	<.001	.176	.015
Meat	% healthy	23%	26%	35%	.537	.148	.031
Fish	% healthy	86%	89%	89%	1.00	.260	.041
Ready-made meals	% healthy	38%	36%	41%	.548	.240	.491
Fried potatoes	% healthy	30%	45%	39%	.003	.316	.066
Rice/noodles	% healthy	86%	82%	86%	.390	.430	1.00
Potatoes	% healthy	66%	66%	65%	.853	.860	.746
Treats^a^	% healthy	42%	22%	43%	< .001	< .001	.879

The categorization of responses into healthy, moderately healthy, and unhealthy is presented in Additional file 1.

t0 = pregnancy, t1 = breastfeeding period, t2 = after weaning.

^a^Treats = combination of sweetened milk products, cakes, and sweet/savory snacks

Regarding the culture of eating, the percentages of participants who reported eating unhealthy snacks between meals were 53% at t0, 63% at t1, and 49% at t2. The median of the number of reported meals per day was 4 at each time point (IQR = 3–4).

### Correlations between nutritional health at t0, t1, and t2

The correlation between the NHS at t0 (pregnancy) and at t1 (breastfeeding) was .55 (*p* < .001), indicating that women who reported a healthier diet during pregnancy also reported a healthier diet in the breastfeeding period. The correlations between the values at t1 and t2 (after weaning) and between the values at t0 and t2 were even stronger (rho = .58 and .60, respectively, *p <* .001).

### Differences between average nutritional health and diet composition at t0, t1, and t2

The overall dietary health of the participating women, as measured using the Nutritional Health Score, was significantly lower in the breastfeeding period than during pregnancy and after weaning (*p* = .005 and .006, respectively). No significant difference was shown between t0 and t2 (*p* = .862). Looking at the consumption levels for the single food items (Table 2), the same pattern (significantly less healthy consumption at t1 than at t0 and t2) was observed for the consumption of treats. For the consumption of sweetened beverages and white bread, the pattern was similar, but only one difference (between t0 and t1 or between t1 and t2) reached statistical significance. Furthermore, the percentage of participants snacking between meals was significantly higher at t1 (63%) than at t0 (53%) or t2 (49%) (*p* = .046 and .007), with no significant differences between t0 and t2 (*p* = .449).

For fried potatoes and unsweetened milk products, in contrast, the percentage of healthy consumption was highest at t1, with at least one difference (either between t0 and t1 or between t1 and t2) reaching statistical significance for each food category. In terms of healthy consumption levels of the other foods (fruits/vegetables, whole grain bread, meat, fish, potatoes, ready-made meals, rice/noodles, and potatoes), we observed no significant differences between t0 and t1 or between t1 and t2. However, the analyses revealed some significant differences between the participants’ diets at t0 and t2. While the consumption levels for fruits/vegetables and white bread were healthier at t0 than at t2, the consumption levels for sweetened beverages, wholegrain bread, meat and fish were healthier at t2 than at t0 (see Table 2).

Regarding the number of reported meals per day, we did not find significant differences between the time points (all *p* > .05).

### Associations between the nutritional health score and—respectively—age, SES, BMI before pregnancy, and previous births

Associations between the NHS and age, SES, BMI before pregnancy, and previous births are presented in Table 3. Higher age was associated with significantly healthier nutrition at t0, i.e., during pregnancy (b = 1.27, *p* = .021): for women aged 25, the average NHS during pregnancy was estimated 41, compared to 60 for 40-year-old women. The association remained significant after adjusting for SES (b = 1.18 (95% CI 0.10, 2.26), *p* = .033). The same pattern was observed at both t1, i.e., in the breastfeeding period, and t2, i.e., after weaning, but the relationship did not reach significance in either case. No significant association was shown between age and differences in the NHS between time points (all *p* > .05).

**Table 3:** Associations between age, SES, BMI before pregnancy, and previous births and nutritional health at t0 (pregnancy), t1 (breastfeeding period), and t2 (after weaning), and with differences in nutritional health between time points (*n* = 110)

		NHS t0	NHS t1	NHS t2	Differences NHS t1-t0	Difference NHS t2-t1	Difference NHS t2-t0
Age	B	1.27	1.19	.98	−.08	−.21	−0.29
	95% CI	0.19, 2.34	−0.13, 2.51	− 0.31, 2.27	−1.18, 1.02	−1.38, 0.97	− 1.33, 0.76
	P	.021	.077	.137	.890	.730	.590
SES (ref = high)	B	−2.15	−7.37	−10.50	− 5.23	−3.12	−8.35
	95% CI	−10.96, 6.66	−17.23, 2.48	−20.33, -0.67	− 13.94, 3.49	−12.48, 6.24	− 16.55, -0.15
	P	.633	.143	.036	.240	.510	.046
BMI before pregnancy^a^	B	−.17	−.80	.27	−.63	1.07	0.44
	95% CI	−0.92, 0.58	−1.82, 0.23	−0.52, 1.07	−1.53, 0.27	0.13, 2.01	−0.43, 1.32
	P	.660	.130	.500	.170	.026	.320
Previous births (ref = no)	B	4.20	−1.46	−1.13	−5.66	0.33	−5.33
	95% CI	−4.43, 12.84	−11.12, 8.21	−11.32, 9.07	− 14.75, 3.43	−9.46, 10.12	−14.00, 3.33
	P	.340	.770	.830	.220	.947	.220

^a^Associations with BMI before pregnancy were assessed in a subsample (*n =* 97) due to missing BMI data for a number of participants

Higher SES was significantly associated with healthier nutrition at t2, i.e., after weaning (b = − 10.50, *p* = .036). The NHS in women of high SES was estimated 56, compared to 45 in women of middle SES. Once age was included as further independent variable, the association no longer reached significance (b = − 9.42 (95% CI -19.22, 0.39), *p* = .060). Similarly, the associations between SES and NHS at t0 and t1 point to a similar variance, but were not statistically significant. A significant association between SES and variation in NHS between t0 and t2 (b = -8.35, *p* = .046) indicated an increase in nutritional health from t0 to t2 (estimated difference = + 4.3) in women of high SES, but a decrease in nutritional health among women of middle SES (estimated difference = − 4.0). This association remained significant after adjusting for age (b = − 8.80 (95% CI -17.08, − 0.51), *p* = .038).

No significant associations were found between higher BMI before pregnancy and NHS at any of the time points (all *p* > .05). Nor was any association measured between the difference in the NHS from t0 to t1 and BMI (*p >* .05). In contrast, there was a significant association between BMI before pregnancy and the difference in the NHS from t1 to t2 (b = 1.07, *p* = .023). For women with a BMI of 18 (underweight), the model estimated a small increase in nutritional health from t1 to t2 (difference = 0.47). With increasing BMI, this increase became more pronounced. For women with a BMI of 23 (normal weight), the estimated difference between t1 and t2 was 5.82, and for women with a BMI of 35 (obese), the estimated increase was 18.65. The associations between BMI before pregnancy and the difference in NHS between t1 and t2 remained significant after adjusting for age and SES (b = 1.19 (95% CI 0.23, 37.33), *p* = .015).

Women with at least one previous birth did not differ from first-time mothers in terms of nutritional health at t0, t1, and t2 (all *p* > .05). Also, the change in diet between t0 and t1 and between t1 and t2 did not differ between the two groups of women (all *p >* .05).

## Discussion

The present study investigated changes in dietary behavior (amount of healthy/unhealthy nutrition) from pregnancy to the period following weaning. The average Nutritional Health Scores of the participating women ranged from approximately 45 in the breastfeeding period to 50 during pregnancy and after weaning. Based on these scores, the overall nutritional health in the present sample can be considered high. However, over three quarters of participants reported unhealthy consumption levels of fruits/vegetables, treats, and meat for at least one of the time points. Overall, women who reported a healthy diet at one time point also reported a healthy diet at the other time points, indicating stability in eating behavior and nutritional health in the period from pregnancy to 1 year after giving birth. However, it is important to note that this period is relatively short and represents a stage of life when one is particularly aware of the importance of healthy eating. It may be that dietary health tends to be poorer and less stable over time at other stages of life [27].

### Differences in diet composition and nutritional health at the different time points

We observed that, overall, the healthiness of the reported diets was significantly lower in the period of breastfeeding than during pregnancy or after weaning. A more detailed look at differences in the consumption of the single food items suggests that this pattern might be best explained by a significantly higher consumption of treats (sweetened milk products, cakes, and sweet or savory snacks) in the breastfeeding period. The frequency of snacking between meals was also significantly higher in the breastfeeding period. Overall, these findings suggest that additional energy requirements in the breastfeeding phase [13] are met primarily by consuming high-calorie and unhealthy foods and less through the consumption of healthier carbohydrates or proteins (e.g., fruits, rice, potatoes, unsweetened milk products, or whole grain products). A possible reason for this is that high-calorie products are easily available (they do not need to be prepared). Three months after giving birth, mothers are often stressed by breastfeeding and childcare and suffer from sleep inefficiency [28]. The initial support provided immediately after birth, such as subsidized household help or parental leave for the father, is often less available during this period, while external childcare options (e.g., daycare centers) are not available until later, at least in Germany. Therefore, during this time, the mother’s own time and energy resources may be directed more towards caring for the child or children and less towards providing for her own nutritional needs.

This finding is concerning given the importance of a healthy diet not only during pregnancy but also in the breastfeeding period. An excessive calorie intake in this period might have negative consequences for the mother (e.g., weight retention [29]) but also for the child (e.g., for metabolic health [5]). Our data indicate that most women resume healthier eating habits after breastfeeding; however, they also suggest that women in the early stages of motherhood need specialized support to maintain a healthy diet. (Expectant) mothers should be made aware of the problem and existing support services (e.g., home delivery of meals, guidebooks) during and shortly after pregnancy, e.g., by gynecologists or midwives.

The analyses also revealed a number of significant differences in dietary healthiness between pregnancy and the period shortly after weaning. While some differences, e.g., concerning meat, fish, wholegrain bread, and sweetened beverages, indicate a healthier diet after weaning than during pregnancy, other differences, e.g. regarding white bread and fruits/vegetables, suggest a healthier diet during pregnancy than after weaning. Therefore, it is not possible to tell whether overall diet is healthier during pregnancy or after weaning. This is in line with the observation of very similar Nutritional Health Scores at both time points.

It should be emphasized that the findings are based on a small and relatively homogeneous sample and a rough categorization of food consumption into (moderately) healthy or unhealthy. As such, the reported differences between the time points may only reflect the most noticeable changes between pregnancy and the period shortly after weaning.

### Associations between nutritional health and—respectively—age, SES, BMI before pregnancy, and previous births

The present findings show that older women report a healthier diet during pregnancy than younger women. The same finding was reported in previous studies [16, 18] and may be explained by higher levels of health awareness as age increases. However, no significant association with age was observed after the birth of the child, either for the breastfeeding phase or the post-weaning period. This finding suggests that the tendency for older women to be more careful to eat healthily is only (or especially) applicable during pregnancy. The association between higher age and an increased risk of complications during pregnancy, e.g., hypertension or pre-eclampsia [30], and adverse pregnancy outcomes, e.g., stillbirths or premature births [31], may lead older women to pay greater attention to maintaining a healthy lifestyle during pregnancy.

In line with previous studies [16–18], we observed a healthier diet in women of high SES compared with women of middle SES. However, this difference only reached significance for the period after weaning, i.e., in the period that might best reflect “normal” life. The difference between SES groups might be explained by differences in the level of investment in maintaining a healthy lifestyle among women of different socio-economic status [32], by differences in the capacity to cope with challenging and new situations (e.g., pregnancy), or by financial limitations, e.g., on buying high-quality food [33]. Interestingly, our data also indicates that the diets of women from lower social strata is healthier during pregnancy than after weaning, while the opposite is observed among women of higher social status. Women of lower SES, who—as we have shown—generally report a less healthy diet than women of higher SES, may make an effort to follow the recommendations for a healthy diet during this important period.

Regarding BMI, our study revealed a significant association between the increase in dietary healthiness from the breastfeeding phase to the period after weaning and higher BMI. This finding might indicate a greater variation in nutritional health among women with a higher BMI. However, the effect was relatively weak and dietary healthiness at t1 and t2 was not, itself, significantly associated with BMI. This latter finding runs counter to both our hypothesis and a previous study that reported a healthier diet in normal-weight women compared to overweight and obese women [17]. A possible explanation for this is that women with a higher BMI tend to underreport their energy intake [34].

With regard to previous births, we did not observe significant differences in nutritional health between first-time mothers and mothers who had given birth previously. This is in line with a previous study [15] and might suggest that neither increased experience and/or confidence, nor additional demands on time, have a decisive influence on nutrition during pregnancy or postpartum.

Overall, it should be noted that a larger and more representative sample might have increased variability in the variables assessed, possibly resulting in stronger associations, e.g., between social status and diet or between BMI and diet.

### Strengths and limitations

To the best of our knowledge, this is the first study to compare the diets of women during pregnancy, in the period of breastfeeding, and after weaning. However, some limitations should be acknowledged. The participants’ diets were only assessed using a short questionnaire. The advantage of this instrument is that it provides a time-effective means of recording nutrition. A disadvantage, however, is the limited accuracy of the dietary assessment. Smaller changes in dietary patterns may remain undetected. The classification of nutrition into healthy, moderately healthy, and unhealthy is practical, but even more imprecise since differences within each category are not taken into account. Another limitation is the relatively small sample size and the resulting lack of statistical power. Also, the study population was not representative in terms of SES, with none of the participating women classed as having low SES. The inclusion of women from lower social strata would have increased the variability in the individual variables, possibly revealing stronger associations. Finally, we have no data on the participants’ diets before pregnancy, nor were we able to assess other potential factors that might be associated with diet during pregnancy, in the breastfeeding period, or after weaning, e.g., the mother and child’s physical or mental health or partner relationship.

## Conclusions

The present study shows that, on average, women consume more treats and snack more frequently between meals during the breastfeeding period than during pregnancy and after weaning. These findings suggest that the additional energy requirements of the breastfeeding phase are mainly met by snacking on unhealthy foods. The result underlines the need to give dedicated support to women in the frequently challenging period shortly after birth and during breastfeeding. This should include not only sleep promotion and help with childcare but also help with the preparation and intake of adequate, healthy food.

## Supplementary Information

**Additional file 1.** Categorization of diet composition based on German dietary guidelines.

**Additional file 2.** Items of CoCu used in the present study.

## Data Availability

The datasets generated and/or analyzed during the current study are not publicly available due to ethical restrictions. The LIFE Child study is a study collecting potentially sensitive information. Publishing data sets is not covered by the informed consent provided by the study participants. Furthermore, the data protection concept of LIFE requests that all (external as well as internal) researchers interested in accessing data sign a project agreement. Researchers that are interested in accessing and analyzing data collected in the LIFE Child study may contact the data use and access committee (forschungsdaten@medizin.uni-leipzig.de).

## Abbreviations

BMI: Body Mass Index
CoCu: Composition and Culture of Eating
NHS: Nutritional Health Score
SES: Socio-economic status
