# Supplementary material for: Changes in diet from pregnancy to one year after birth: a longitudinal study
Source: BMC Pregnancy Childbirth. 2021 Sep 4;21:600. doi: 10.1186/s12884-021-04038-3 (PMC8418026; doi:10.1186/s12884-021-04038-3)
Supplement: Supplementary file 1 — Additional file 1. Categorization of diet composition based on German dietary guidelines. [file 12884_2021_4038_MOESM1_ESM.pdf]

**Additional file 1**

**Categorization of diet composition based on German dietary guidelines**

Article: Changes in diet from pregnancy to one year after birth: a longitudinal study

Authors: Tanja Poulain, Ulrike Spielau, Mandy Vogel, Anne Dathan-Stumpf, Antje Körner, Wieland Kiess

Journal: BMC Pregnancy and Childbirth

| Colour | Meaning |
|---|---|
| Green | Healthy |
| Yellow | Moderately healthy |
| Red | Unhealthy |

| | Response categories | | | | | |
|---|---|---|---|---|---|---|
| | 0 portions | max. 1 portion | 2-3 portions | 4-5 portions | 6-7 portions | > 7 portions |
| **Consumption per day** | | | | | | |
| Fruits/vegetables | -10 points | -10 points | 0 points | 10 points | 10 points | 10 points |
| Unsweetened milk products | -10 points | 0 points | 10 points | 0 points | -10 points | -10 points |
| Sweetened beverages | 10 points | 0 points | -10 points | -10 points | -10 points | -10 points |
| Wholegrain bread | -10 points | 0 points | 10 points | 0 points | -10 points | -10 points |
| White bread | 10 points | 10 points | 0 points | -10 points | -10 points | -10 points |
| | | | | | | |
| **Consumption per week** | | | | | | |
| Meat | 0 points | 0 points | 0 points | 10 points | 0 points | -10 points |
| Fish | 0 points | 10 points | 10 points | 0 points | -10 points | -10 points |
| Ready-made meals | 10 points | 0 points | -10 points | -10 points | -10 points | -10 points |
| Fried potatoes | 10 points | 0 points | -10 points | -10 points | -10 points | -10 points |
| Potatoes | -10 points | 0 points | 10 points | 10 points | 10 points | 10 points |
| Rice/noodles | -10 points | 0 points | 10 points | 10 points | 0 points | 0 points |

For "treats" (sum of weekly portions of sweetened milk products, cakes, and sweet/savory snacks), up to 7 portions/week are categorizes as "green", between 8 and 11 portions/week are categorized as "yellow", and ≥ 12 portions/week are categorized as "red".

Reference: Kersting M, Alexy U, Clausen K. Using the concept of Food Based Dietary Guidelines to develop an Optimized Mixed Diet (OMD) for German children and adolescents. J Pediatr Gastroenterol Nutr. 2005;40:301–8.
