# Supplementary material for: Changes in diet from pregnancy to one year after birth: a longitudinal study
Source: BMC Pregnancy Childbirth. 2021 Sep 4;21:600. doi: 10.1186/s12884-021-04038-3 (PMC8418026; doi:10.1186/s12884-021-04038-3)
Supplement: Supplementary file 2 — Additional file 2. Items of CoCu used in the present study. [file 12884_2021_4038_MOESM2_ESM.pdf]

Additional file 2

Items of CoCu used in the present study

Article: Changes in diet from pregnancy to one year after birth: a longitudinal study

Authors: Tanja Poulain, Ulrike Spielau, Mandy Vogel, Anne Dathan-Stumpf, Antje Körner, Wieland Kiess

Journal: BMC Pregnancy and Childbirth

Original version (German)

**Fragen zur Nahrungszusammensetzung**[a]	
Wie viele … essen Sie PRO TAG?	
1	Portionen Obst und/oder Gemüse (1 Portion = 1 Apfel, 1 Birne, 1 Kohlrabi)[b]
2	Portionen ungesüßter Milchprodukte (1 Portion = 1 Glas Milch, 150g Quark, 150g Naturjoghurt, 1 Scheibe Käse)
3	Portionen gesüßter Milchprodukte (1 Portion = 150g Pudding, 150g Fruchtjoghurt, 150g Fruchtquark, 1 Glas Kakao)
4	Gläser süßer Getränke (z.B. Limonade, Saft, gesüßter Tee oder Kaffee, Wasser mit Geschmack)
5	Portionen Vollkornbrot/Vollkornbrötchen (1 Portion = 1 Scheibe bzw. 1 Brötchen)
6	Portionen Weißbrot/Mischbrot/Toastbrot/helle Brötchen (1 Portion = 1 Scheibe bzw. 1 Brötchen)
Wie viele … essen Sie PRO WOCHE?	
7	Portionen Fleisch und Wurst (1 Portion = 3 Scheiben Wurst) [b]
8	Portionen Fisch (1 Portion = 1 Dosenfisch, 3 Fischstäbchen)[b]
9	Portionen Fertiggerichte (z.B. Tiefkühlpizza, Tiefkühllasagne, Nudeltöpfe, Mikrowellengerichte)[b]
10	Portionen Salz- oder Pellkartoffeln[b]
11	Portionen Pommes und/oder Kroketten und/oder Bratkartoffeln[b]
12	Portionen Reis/Nudeln[b]
13	Portionen Kuchen, Torten, Kekse, Gebäck (1 Portion = 1 Stück Torte/Kuchen, 3-4 Stück Kekse)
14	Portionen Süßes und/oder Salziges (1 Portion = 1 Schokoriegel)[b]
**Fragen zur Esskultur**	
15	Welche Mahlzeiten essen Sie pro Tag? [c]
16	Naschen Sie gewöhnlich zwischen den Mahlzeiten?[d]

[a] Antwortoptionen = nie; max. 1; 2-3; 4-5; 6-7; >7 Portionen

[b] Fotos von Referenzportionen dienen als zusätzliche Einschätzungshilfe

[c] Antwortoptionen (Mehrfachantworten möglich): Frühstück; 2. Frühstück; Mittagessen; Nachmittagsmahlzeit; Abendessen

[d] Antwortoptionen: ja; nein

English translation

**Diet composition part**[a]	
How may … do you eat PER DAY?	
1	Portions of fruit/vegetables (1 portion = 1 apple, 1 pear, 1 kohlrabi)[b]
2	Portions of unsweetened milk products (1 portion = 1 glass of milk, 150 g quark, 150 g natural yoghurt, 1 slice of cheese)
3	Portions of sweetened milk products (1 portion = 150 g pudding, 150 g fruit yoghurt, 1 cup of cocoa)
4	Glasses of sweetened beverages (e.g., lemonade, sweetened tea, flavored water)
5	Portions of wholegrain bread/roll (1 portion = 1 slice of bread, 1 roll)
6	Portions of white bread/brown bread/toast/white roll (1 portion = 1 slice of bread, 1 roll)
How may … do you eat PER WEEK?	
7	Portions of meat/sausage (1 portion = 3 slices of sausage)[b]
8	Portions of fish (1 portion = 1 canned fish, 3 fish fingers)[b]
9	Portions of ready-made meals (e.g., frozen pizza, frozen lasagne, instant noodles, microwave meals)[b]
10	Portions of boiled potatoes/potatoes cooked in skins[b]
11	Portions of fried potatoes (e.g., fries, croquettes, roast potatoes)[b]
12	Portions of rice/noodles[b]
13	Portions of cakes/cookies/pastries (1 portion = 1 piece of cake, 3-4 cookies)
14	Portions of sweet or savory snacks (1 portion = 1 chocolate bar)[b]
**Culture of eating part**	
15	Which meals do you eat each day?[c]
16	Do you usually eat unhealthy snacks between meals?[d]

[a] Response options = never; max. 1; 2-3; 4-5; 6-7; >7 portions

[b] Photographs of reference portions are provided as an additional guide.

[c] Response options (multiple responses possible): first breakfast; second breakfast; lunch; afternoon snack; dinner

[d] Response options: yes; no

Reference: Poulain T, Spielau U, Vogel M, Körner A, Kiess W. CoCu: A new short questionnaire to evaluate diet composition and culture of eating in children and adolescents. Clin Nutr. 2019;38:2858–65.
